# Improving restoration heuristics to support anadromous fish passage

**DOI:** 10.1371/journal.pone.0348150

**Published:** 2026-06-03

**Authors:** Sunny L. Jardine, Logan Blair, Catalina Burch, Andrew Cooke, Robert Fonner, Daniel S. Holland, J. Kahn, Connor Lewis-Smith, Luke W. Rogers, Mark D. Scheuerell, Braeden Van Deynze

**Affiliations:** 1 School of Marine and Environmental Affairs, University of Washington, Seattle, Washington, United States of America; 2 Washington State Department of Ecology, Lacey, Washington, United States of America; 3 Center for International Trade in Forest Products, University of Washington, Seattle, Washington, United States of America; 4 Northwest Fisheries Science Center, National Marine Fisheries Service, NOAA, Seattle, Washington, United States of America; 5 Quantitative Ecology and Resource Management, University of Washington‌‌, Seattle, Washington, United States of America; 6 U.S. Geological Survey Washington Cooperative Fish and Wildlife Research Unit, School of Aquatic and Fishery Sciences, University of Washington, Seattle, Washington‌‌, United States of America; 7 Washington Department of Fish and Wildlife, Olympia, Washington‌‌, United States of America; Universität für Bodenkultur Wien: Universitat fur Bodenkultur Wien, AUSTRIA

## Abstract

Investments in restoring river connectivity are growing worldwide to support freshwater biodiversity. Although optimization methods exist for selecting cost-effective restoration portfolios, decisions are often guided by simple heuristic rules. For example, managers may prioritize restoring barriers blocking the largest amounts of high-quality upstream habitat, ignoring the position of other barriers in the system. These heuristics often rely on proxies for watershed connectivity and habitat quality. Using anadromous fish passage restoration in western Washington, USA, as a case study, we show that redesigning these heuristics can yield substantial performance gains. Benchmarking common heuristics against optimization outcomes reveals that connectivity proxies based on total upstream habitat can achieve 93% of optimal gains when increasing habitat quantity is the sole objective, but adding widely used proxies for habitat quality (e.g., percent of upstream natural land cover) can cut performance nearly in half. These findings underscore the importance of designing heuristics that more directly target high-quality habitat gains to improve investment efficiency and help close the science–practice gap between optimization research and on-the-ground restoration decisions.

## 1 Introduction

River fragmentation is a global problem [1–3], widely recognized as a major threat to freshwater biodiversity [4,5] and the socioeconomic benefits provided by freshwater species, such as food security [6]. While hydropower dams contribute to river fragmentation [7,8], smaller barriers such as culverts, fords, and artificial impoundments are far more numerous in many river systems [2,9,10]. As awareness of the ecological and socioeconomic impacts of barriers to fish passage grows, investments in restoring river connectivity are also rising. Examples include over $1 billion in fish passage grant programs included in the United States Bipartisan Infrastructure Law and Inflation Reduction Act [11] and the European Union commitment to restoring connectivity in 25,000 km of rivers by 2030 [12]. Thus, the efficiency of investments in fish passage restoration is ever more important.

Heuristic decision rules, defined as “methods for arriving at satisfactory solutions with modest amounts of computation” [13], are widely used in conservation decision-making. The conservation targeting literature has examined the effectiveness of various heuristic strategies for allocating limited resources to conservation, particularly in land protection [14–17]. This body of work has explored benefit targeting approaches, such as score-and-rank methods, as well as cost targeting strategies [18]. However, much of this research assumes that the benefits and costs of each project alternative are constant over time and independent across sites, with interdependencies primarily considered in the context of agglomeration benefits [19] and land market dynamics [20].

**Fig 1:**
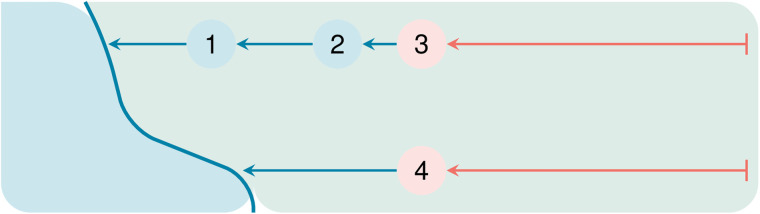
Stranded investments can occur when ignoring project connectivity in the presence of upstream and downstream barriers. Schematic of two stream networks simplified as lines (actual networks are dendritic without loss of generality), with barriers to fish passage shown as numbered circles. The large blue polygon represents the ocean, making barrier 1 the most downstream barrier in the system. Blue barriers and stream segments indicate restored barriers and accessible habitat, respectively, while red barriers and stream segments indicate impediments to fish passage and blocked habitat. A downstream-to-upstream strategy that targets barriers with the most upstream habitat (e.g., restoring barriers 1–2) can produce stranded investments, as gains from barrier 2 are constrained by barrier 3. An optimization approach instead selects barriers 1 and 4, maximizing total habitat gains across both networks.

In contrast, fish passage restoration presents a unique challenge because its effectiveness depends on watershed connectivity, i.e., the removal of one barrier alone may not yield benefits unless other barriers in the network are also addressed. This introduces the possibility of stranded investments, where projects fail to deliver their intended ecological outcomes due to unaddressed upstream or downstream barriers (see Fig 1). Although heuristics are commonly used in fish passage restoration [21,22], existing evaluations of their performance have largely focused on cases where benefits are well defined, i.e., clearly connected to restoration goals, and watershed connectivity is ignored. This literature finds substantial gains from switching from a heuristic to an optimization approach in selecting fish passage restoration portfolios [23–25].

**Fig 2:**
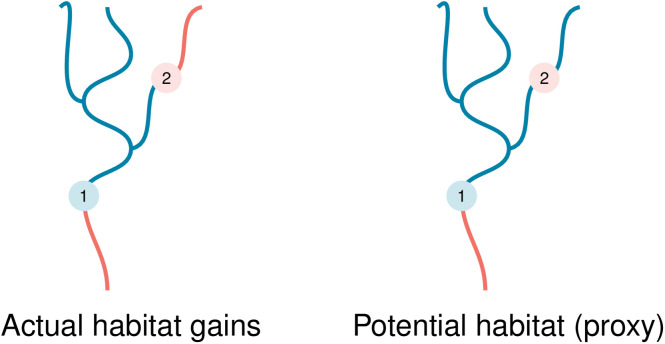
Illustration of actual (left) versus proxy-based (right) habitat gains for barrier removal. Both panels show the same simplified river network with two barriers: barrier #1 (blue marker) and an upstream barrier, barrier #2 (pink marker). In each panel, blue stream segments denote habitat that is counted as accessible following removal of barrier #1 under the specified metric, while red stream segments denote all remaining habitat in the network that is not counted toward that metric (including downstream habitat). With actual habitat gain (left panel) removing barrier #1 restores access only to habitat upstream of barrier #1 and downstream of any remaining upstream barriers (here, barrier #2). Potential habitat gains are a common alternative metric (right panel) which proxy for watershed connectivity by counting all habitat upstream of barrier #1, ignoring the presence of any upstream barriers such as barrier #2.

However, the existing literature does not shed light on the performance of contemporary approaches to fish passage restoration, which often attempt to incorporate watershed connectivity and are rarely based on clearly defined benefits. Instead, many current heuristic methods rely on proxy metrics to adjust for, rather than directly account for, both watershed connectivity and desired habitat quality features. Examples include using “potential habitat” rather than actual habitat as a proxy for watershed connectivity (Fig 2) and the percentage of a barrier’s upstream habitat comprised of natural land cover as a proxy for the goal of increasing stream habitat surrounded by natural land cover. These features, i.e., proxies for connectivity and habitat quality, are shared by fish passage heuristics developed in systems around the world to promote the recovery of migratory species, including alewife, blueback herring, eel, lamprey, salmon, and trout [21,26–29]. Despite significant investments in these systems, the performance of these heuristics relative to optimization methods remains unexamined.

While the science of selecting fish passage restoration projects to maximize return on investment is well established, [e.g., 8,23], the adoption of optimization approaches in practice has been limited [9]. Instead, decision-making often relies on heuristics or simple, rule-based strategies that persist despite documented limitations. This continued reliance reflects what is commonly referred to as the science-practice gap [30,31]. Yet heuristics offer notable advantages in their flexibility, transparency, and ease of implementation, making them especially valuable in real-world restoration planning contexts [22,32,33]. In this study, we focus on evaluating the performance of existing heuristics and exploring their potential for improvement. The specific research questions guiding this work are: (1) How do current heuristics perform relative to optimization benchmarks?; and (2) Are optimization approaches needed for large performance gains, or can a less invasive redesign of current heuristics deliver meaningful improvements? Using fish passage restoration for anadromous salmon and steelhead in Western Washington as a case study, we quantify the benefits of narrowing the science-practice gap through more effective heuristic design. Our findings highlight specific heuristic features that enhance investment outcomes and demonstrate how targeted refinement, rather than wholesale replacement by optimization, can support more strategic and impactful restoration investments.

## 2 Methods and materials

### 2.1 Study area

**Fig 3:**
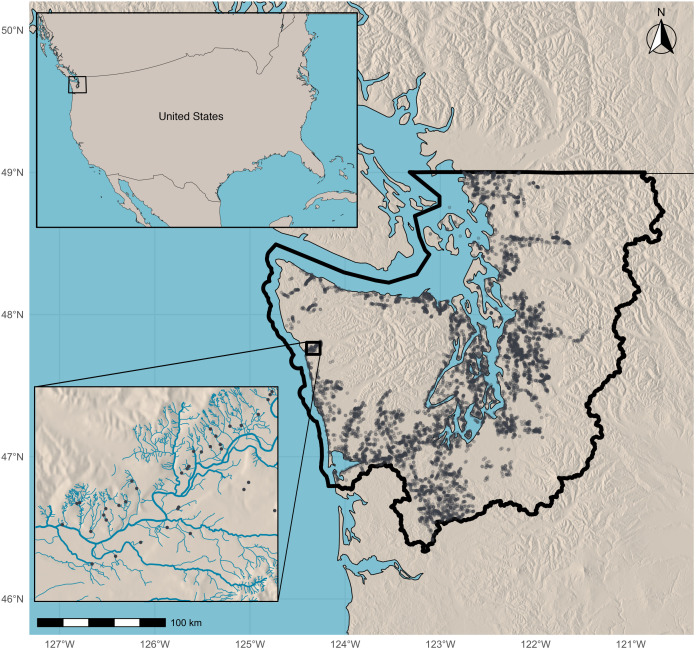
Map of the Case Area of Western Washington, US. Barrier culverts are points on the map and the inset shows culverts on a section of the Hoh river. Culvert locations are from Washington Department of Fish and Wildlife (WDFW) Fish Passage Inventory. The Case Area boundary is taken from the Washington State Department of Transportation (WSDOT) and shown with a thick black line. Basemap sources include: Natural Earth (country borders), and the U.S. National Aeronautics and Space Administration (NASA) Shuttle Radar Topography Mission (SRTM) elevation data (shaded relief).

We focus on the example of restoring access to salmon habitat in the Western Washington region of the United States (Fig 3), through the removal of culverts blocking fish passage. In 2018, in what has been referred to as the “Culverts Case”, the United States Supreme Court affirmed that culverts blocking salmon from accessing upstream habitat are in violation of tribal treaty fishing rights [34]. As a remedy, the courts issued a permanent injunction requiring that the state of Washington remove barrier culverts under its jurisdiction in the “Case Area,” the lands and waters ceded by the Tribes party to the lawsuit against the state, such that 90% of blocked fish habitat is made accessible by 2030. Fig 3 shows the Case Area and its barrier culverts. The inset illustrates a section of the Hoh River, highlighting how some barrier culverts do not align with mapped stream lines, an important issue for data processing described below.

In the Washington Case Area, all entities engaged in removing barriers to fish passage currently use a multi-objective “score-and-rank” (SR) heuristic approach to prioritization. These approaches integrate proxy metrics for both connectivity and habitat quality and are widely used because they are practical and adaptable to diverse management contexts [21]. However, systematic evaluation of their performance using optimization benchmarks is needed and is the goal of this analysis.

### 2.2 Data sources

We focus on 6,773 artificial barriers that we determine to be potential restoration projects (Fig 3). We compile project attributes from five primary sources: the USGS National Hydrography Dataset plus attributes in high resolution (NHDPlus HR), the Washington Department of Fish and Wildlife (WDFW) Fish Passage Barriers Inventory downloaded April 18, 2024 (hereafter the “WDFW inventory”), the NorWeST Stream Temperature Regional Database [35], the 2021 National Land Cover Database (NLCD), and a predictive cost model from [36] trained on over 1,200 completed culvert projects in Oregon and Washington, documented in the Pacific Northwest Salmonid Habitat Projects (PNSHP) dataset [37].

### 2.3 Data processing

The NHDPlus HR stream network is first cleaned to remove non-habitat features, including coastal lines, artificial paths, canals, ditches, pipelines, underground conduits. We also remove network divergences to create a dendritic stream system, required for optimization, following [23]. Barriers from the WDFW inventory matched to removed divergences were excluded (the matching procedure is described below), leaving 22,001 natural and artificial barriers.

Next, each of the 22,001 natural and artificial barriers is matched to the nearest NHDPlus HR stream using a two-step process. First, we manually classify a sample of 301 culverts to determine whether each is located on a streamline represented in the NHDPlus HR database. Using this labeled sample, we train a random forest model that predicts whether a given barrier is correctly matched to its nearest NHDPlus HR stream. The model incorporates variables describing spatial relationships between barriers and lidar-derived streamlines and mapped roads, as well as the culvert’s cross-sectional area, streamflow attributes of the closest NHDPlus streamline, and the similarity in stream names between the WDFW inventory and the NHDPlus database. The model achieves F1-scores of 0.91 for correctly matched barriers and 0.94 for incorrect matches. We then apply fuzzy name matching, using names from the WDFW inventory and the NHDPlus database, to further refine predictions, resulting in 9,729 culverts classified as well matched to their NHDPlus HR stream.

Culverts located upstream of non-culvert barriers (e.g., waterfalls or dams) are removed, leaving 6,925 barriers eligible for restoration. Restoration cost estimates are assigned using predictions from the [36] model adjusted for inflation. For barriers added to the inventory after the [36] predicted cost dataset was generated, we interpolate cost using inverse distance weighting of up to 10 neighboring points. Finally, barriers located so close in proximity to each other that their upstream–downstream relationship cannot be determined are combined into single projects, summing predicted costs, producing a final dataset of 6,773 unique potential restoration projects.

### 2.4 Benefit metrics

For each project, we calculate a suite of benefit metrics that together capture both the quantity and quality of habitat restored (Table 1). These metrics are representative of approaches used at our study site and other fish passage restoration programs [21,26–29] detailed in Table SI1 in S1 File.

**Table 1:** Metric inputs to barrier scores.

Objective	Benefit Accounting	Benefit Proxy	Source
Total Habitat	Gain in total habitat or habitat to the next upstream barrier(s) if any (km)	Habitat potential or total upstream habitat (normalized)^†^	USGS NHDPlusHR
Natural Habitat	Gain in habitat surrounded by natural land cover to the next upstream barrier(s) if any (km)	Percent of upstream habitat that is natural land cover (%)^‡^	USGS National Land Cover Dataset
Cool-water Habitat	Gain in habitat with cool stream temperatures to the next upstream barrier(s) if any (km)	Deviation of upstream water temperature from a desired temperature (normalized)^‡^	NorWeST Database
Species Habitat	Gain in habitat for target species to the next upstream barrier(s) if any (km)	Count of target species that potentially benefit from the project (normalized)^‡^	WDFW Inventory

Variables denoted with ^†^ are proxies for connectivity and variables denoted with ^‡^ are proxies for habitat quality. Proxies are representative of those used in our study area and around the world (see Table S1 in S1 File).

We begin with habitat quantity. Linear habitat gains from removing barrier *j*, denoted $h_{j}^{a}$, are calculated as the length of habitat between barrier *j* and the nearest upstream barrier(s), or to the headwaters if no upstream barrier exists. These represent the *actual* gains from removing barrier *j*, illustrated in the left panel of Fig 2. To account for watershed connectivity, common heuristics replace actual gains with total *potential* upstream habitat, $h_{j}^{\rho }$, which includes all habitat upstream of *j* while ignoring any upstream barriers; this proxy is widely used, as documented in nine programs in the US and Australia (Table SI1 in S1 File), and is illustrated in the right panel of Fig 2.

Habitat quality is then assessed along each stream segment. We quantify the proportion of habitat within a 100 m buffer around the stream line that is classified as natural land cover (barren, forest, herbaceous, shrubland, or open water) using the 2021 National Land Cover Database (NLCD). This produces fractions of actual ($n_{j}^{a}$) and potential ($n_{j}^{p}$) natural habitat, surrounded by natural land cover, which serve as inputs for both direct benefit calculations, where the fractions may be multiplied by the length of the stream segment ($h_{j}^{a}$ or $h_{j}^{\rho }$) to obtain actual or potential natural habitat in kilometers, or used directly in some heuristic algorithms (see Table 1). We note that the fraction of natural land cover surrounding a barrier or upstream of it is a common metric for habitat quality, and in this context it serves as a proxy for the underlying management goal of increasing salmon habitat that is embedded within naturally vegetated landscapes [21].

Thermal suitability of habitat is another common management goal and is captured using NorWeST August temperature predictions (1993–2001), representing the peak temperatures experienced by anadromous species [38]. Missing values are interpolated via inverse distance weighting of up to 10 neighboring points, weighted by bankfull width. We define cool-water habitat as a binary indicator, i.e., $t_{j}^{r}=1$ when upstream temperatures fall within the optimal coho salmon range (8.5–18°C), from [39], and $t_{j}^{r}=0$ otherwise. Deviations from the desired temperature *t*_opt_ = 12.35°C, from [39] and [40], are a common proxy for thermal suitability and are calculated as $\delta_{j}=\exp(-0.1[t_{j}-t_{opt}])$.

Finally, species-specific benefits are quantified using the WDFW inventory, which identifies potential species inhabiting streams blocked by each project *j*. We consider seven species of interest, all of which are anadromous in our study area: bull trout (*Salvelinus confluentus*), cutthroat trout (*Oncorhynchus clarkii*), Chinook (*Oncorhynchus tshawytscha*), chum (*Oncorhynchus keta*), coho (*Oncorhynchus kisutch*), sockeye (*Oncorhynchus nerka*), steelhead (*Oncorhynchus mykiss irideus*), denoting the set of species benefiting from the removal of barrier *j* as *s*_*j*_ and their count as $\left|s_{j}\right|$.

### 2.5 Prioritization Approaches and Benchmarks

**Fig 4:**
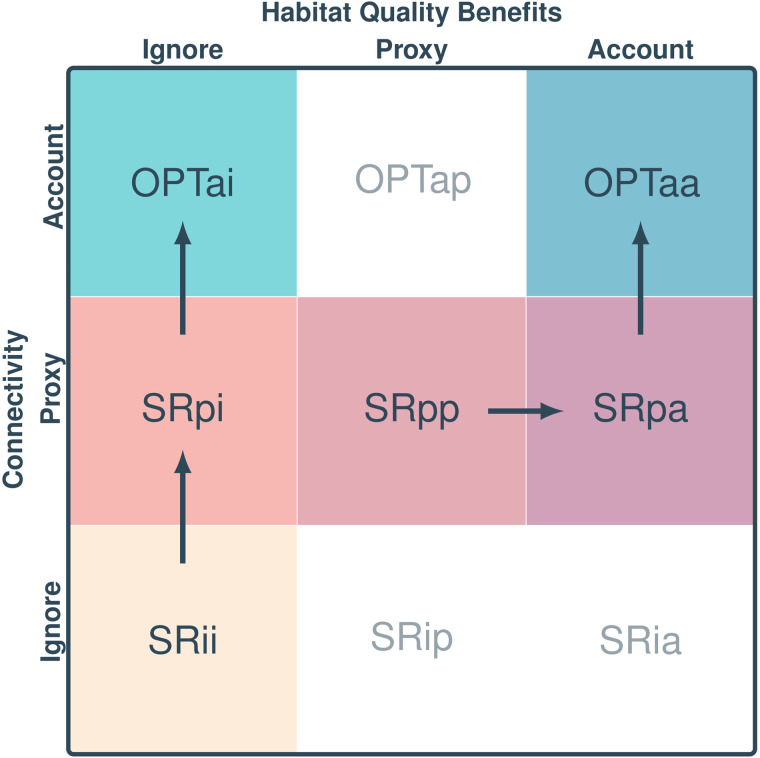
Prioritization approaches and Pathways for Improvement Depending on the Objective Function. Shading indicates whether a scenario is included in the analysis, while each color identifies a specific scenario. Each cell contains a label describing the scenario: the first part indicates the method (optimization, OPT, or score-and-rank, SR), and the two subscripts indicate how connectivity and habitat quality are treated, respectively (with options being ignored, i; proxied, p; or fully accounted for, a). The six scenarios considered here include three quantity-focused approaches (first column) and three quality-focused approaches (second and third columns). Arrows represent pathways to narrowing the science-practice gap, depending on the objective function (i.e., whether habitat quality is a goal). Unshaded cells represent scenarios not included in the analysis.

We evaluate a SR heuristic that integrates proxies for watershed connectivity and habitat quality, alongside several benchmark approaches (see Table 2 and Fig 4). We use the notation METHODcq to describe each approach, where METHOD indicates either optimization (OPT) or score-and-rank (SR), and indices c and q indicate how connectivity and habitat quality are respectively treated with options including: ignored (i), proxied (p), or fully accounted for (a). For example, SRpp represents a score-and-rank heuristic incorporating proxies for both connectivity and habitat quality.

**Table 2:** Summary of prioritization approaches evaluated.

Scenario	Method	Connectivity	Habitat Quality	Notes
SRii	SR	Ignored	Ignored	Quantity-focused
SRpi	SR	Proxy	Ignored	Quantity-focused
OPTai	OPT	Accounted	Ignored	Quantity-focused
SRpa	SR	Proxy	Accounted	Quality-focused
SRpp	SR	Proxy	Proxy	Quality-focused
OPTaa	OPT	Accounted	Accounted	Quality-focused

To isolate the effects of common proxies, we consider quantity-focused benchmarks where habitat quality benefits are ignored. These include:

- SRii: score-and-rank ignoring both connectivity and habitat quality;
- SRpi: score-and-rank using a proxy for connectivity while ignoring habitat quality;
- OPTai: optimization explicitly accounting for connectivity while ignoring habitat quality.

We then examine quality-focused approaches where both habitat quantity and quality are valued:

- SRpa: score-and-rank proxying for connectivity and explicitly accounting for habitat quality;
- SRpp: score-and-rank using a proxy for both connectivity and habitat quality;
- OPTaa: optimization explicitly accounting for both connectivity and habitat quality.

### 2.6 Scoring methods

We develop scores for each of the six approaches (shaded cells in Fig 4) based on the benefit metrics described above. For quantity-focused approaches, scores (*v*) depend solely on habitat gains:


$$\begin{array}{c} v_{j}^{OPTai}=h_{j}^{a},\;v_{j}^{SRpi}=h_{j}^{\rho },\;v_{j}^{SRii}=h_{j}^{a}. \end{array}$$

For quality-focused approaches, scores combine habitat quantity with habitat quality, thermal suitability, and species-specific benefits:


$$\begin{array}{c} v_{j}^{OPTaa}=\alpha h_{j}^{a}+\theta (h_{j}^{a}n_{j}^{a}+h_{j}^{a}t_{j}^{r}+h_{j}^{a}|s_{j}|/S), \end{array}$$

$$\begin{array}{c} v_{j}^{SRpp}=\alpha {\tilde{h}}_{j}^{\rho }+\theta (n_{j}^{p}+\delta_{j}+|s_{j}|/S), \end{array}$$

$$\begin{array}{c} v_{j}^{SRpa}=\alpha h_{j}^{\rho }+\theta (h_{j}^{\rho }n_{j}^{p}+h_{j}^{\rho }t_{j}^{r}+h_{j}^{\rho }|s_{j}|/S). \end{array}$$

Here, $h_{j}^{a}$ and $h_{j}^{\rho }$ denote actual and potential habitat gains, $n_{j}^{a}$ and $n_{j}^{p}$ the corresponding fractions of natural land cover, $t_{j}^{r}$ the cool-water indicator, $\delta_{j}$ the thermal suitability metric, $\left|s_{j}\right|$ the number of species benefiting from barrier removal, and S is the total number of species of interest. A tilde denotes a normalized variable (e.g., ${\tilde{h}}_{j}^{\rho }$). The parameter α is the weight placed on habitat quantity, and θ=(1−α)/3 gives the weight placed on each habitat-quality component. Reflecting the median weight on habitat quantity in our study system [21], we set α=0.3, giving slightly more weight to habitat quantity while still incorporating natural habitat, thermal considerations, and species-specific benefits. In a SR framework, this corresponds to allocating up to 30 points for habitat length and up to 23.3 points each for natural land cover, cool-water suitability, and benefits to target species. Sensitivity analyses on α are reported in the supplementary information (Figure SI3).

### 2.7 Score-and-rank (SR)

The SR framework constructs a restoration portfolio as follows:

1. Compute scores for all barriers
2. Sort barriers from highest to lowest score
3. Initialize the remaining budget to the total budget *B*
4. For each barrier in ranked order:
  (a) If the barrier’s cost is less than or equal to the remaining budget, select it and subtract its cost from the remaining budget
  (b) Otherwise, skip it and move to the next barrier
5. Continue until all barriers have been considered or the remaining budget cannot cover any of the remaining barriers

Note that in this score-and-rank approach, scores are calculated once at the beginning and are not updated after each selection. Because the approach uses habitat potential, which ignores the presence of other barriers, removing a barrier does not alter the value of remaining barriers, so iterative score updates are unnecessary.

### 2.8 Optimization

The optimization problem seeks to maximize total benefits from selected barrier projects while satisfying budget and connectivity constraints. The binary decision variable *x*_*j*_ indicates whether project *j* is selected. Budget constraints require that total expenditures do not exceed the total budget, *B*, while connectivity constraints require that any upstream barrier can only be restored if all barriers directly downstream are also restored.


$$\begin{array}{c} \text{maximize:}\;\sum_{j}v_{j}x_{j} \end{array}$$ (1)

$$\begin{array}{c} \text{subject to:}\;\sum_{j}c_{j}x_{j}\leq B \end{array}$$ (2)

$$\begin{array}{c} x_{j}-\sum_{k}d_{jk}x_{j}\leq 1-\sum_{k}d_{jk},\;\forall j \end{array}$$ (3)

We solve this mixed-integer linear program using Gurobi Optimizer [41].

### 2.9 Evaluative criteria

We compare outcomes from SR approaches to those from exact optimization using four implied restoration goals, which represent actual benefits rather than the proxy metrics used in prioritization. These goals are: total habitat gains; habitat gains within natural land cover; cool-water habitat gains in the optimal temperature range for coho salmon; and habitat gains weighted by the fraction of seven target species benefiting from the removal of all selected projects.

## 3 Results

When maximizing habitat quantity is the only goal, the SR heuristic performs remarkably well. The outcome hinges on the effectiveness of a common practice of adjusting benefits for watershed connectivity considerations (Fig 2). The *actual* habitat quantity benefit of restoring a given project *j*, denoted $h_{j}^{a}$, is calculated as the total length of habitat in all stream segments upstream of *j* that are accessible before encountering the next barrier(s) or the headwaters. As a proxy, benefits can be redefined as the total habitat upstream of *j*, ignoring other barriers in the system, denoted as $h_{j}^{\rho }$. The proxy metric is, therefore, higher for barriers further downstream, generating solutions that generally proceed from downstream up (Fig 2).

**Fig 5:**
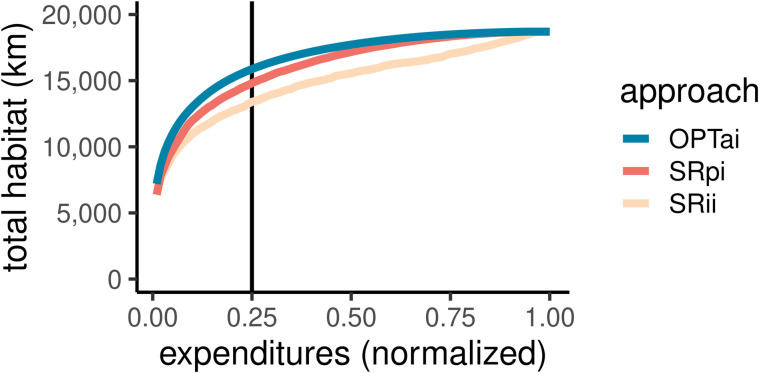
The relationship between normalized expenditures and habitat gains when habitat quantity is the sole objective by approach. Approaches include: accounting for connectivity through optimization (OPTai), using a connectivity proxy (SRpi), and ignoring connectivity (SRii).

Without connectivity adjustments, at a budget level of 25% of the cost to restore all barriers in the system, total habitat generated through the SR approach (SRii) is only 84% of that obtained from optimization (Fig 5). However, the connectivity proxy goes a long way towards closing the gap, achieving 93% of total habitat gains from optimization (Fig 5). This success occurs despite the potential issues associated with a quantity-focused SR approach, such as stranded investments and otherwise inefficient spending. Fig 1 presents a stylized example of how stranded investments can occur with heuristic approaches to anadromous fish passage restoration. The supplementary information further describes inefficient spending (Figure SI1).

**Fig 6:**
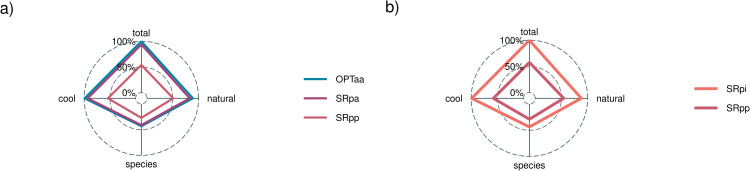
Gains in habitat quantity and high-quality habitat. (a) compares quality-focused approaches (OPTaa, SRpa, SRpp). (b) compares SR approaches ignoring (SRpi) or proxying (SRpp) for habitat quality. Notes: outcomes are from a budget equal to 25% of total cost. For each panel, the 100% line is set to the level of total habitat obtained from the best-performing method.

Importantly, we find that including habitat quality considerations, in the form of benefit proxies, greatly diminishes the heuristic performance. Specifically, when habitat quantity along with multiple dimensions of habitat quality are valued, SRpp substantially underperforms all other prioritization schemes considered, along every dimension of valued restoration attributes (Panel a; Fig 6). For example, as compared to OPTaa, SRpp generates only a fraction of total habitat (54%), natural habitat (57%), cool-water habitat (54%), and habitat for species of interest (63%). Thus, while proxies for connectivity perform relatively well (generating 93% of possible gains), when connectivity proxies are combined with proxies for habitat quality, performance drops sharply, yielding only 54–63% of the potential gains. The diminished performance of SRpp is largely robust across independent watersheds in our study system (Figure SI2), is further reduced at smaller budget levels and when a smaller weight is placed on habitat quantity (Figure SI3) and is not mitigated by common penalties intended to further adjust for watershed connectivity (Figure SI4).

Unsurprisingly, OPTaa, which explicitly accounts for both project benefits and watershed connectivity, performs best in achieving every restoration goal (Panel a; Fig 6). The greatest gains in moving away from SRpp come from redefining project benefits so that they are better aligned with restoration goals. For example, targeting gains in stream habitat surrounded by natural land cover rather than targeting the percentage of a barrier’s upstream habitat comprised of natural land cover. This is evidenced by the fact that SRpa achieves near-optimality, generating 93–95% of gains from OPTaa for all objectives (Panel a; Fig 6) and we find that the improved performance of SRpa over SRpp hold across independent watersheds in our study system (Figure SI2).

**Fig 7:**
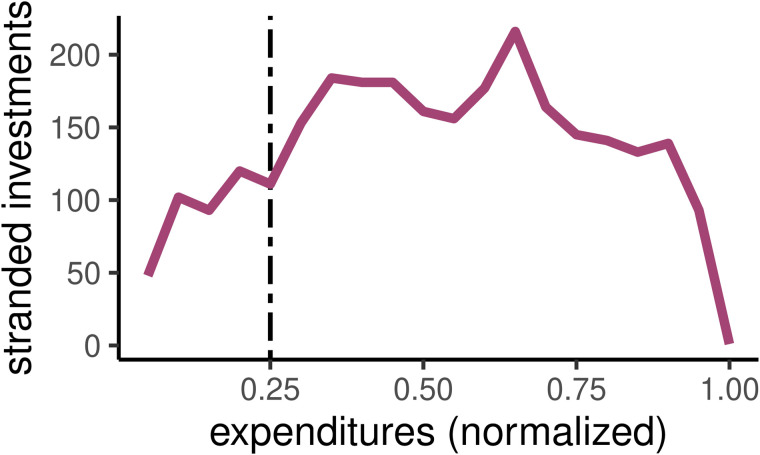
The relationship between (normalized) expenditures and the number of stranded investments from a quality-focused SRpp approach.

What is surprising is the fact that, not only does the SRpp underperform the two other quality-focused approaches, but it also underperforms SRpi, the quantity-focused SR approach that ignores habitat quality entirely (Panel b; Fig 6). For example, including proxies for natural habitat and species of interest *reduces* the resulting gains in these restoration outcomes. This counter-intuitive result can be explained by examining the number of stranded investments generated with the SRpp approach (Fig 7), specifically the number of barriers selected for restoration with unrestored downstream barriers preventing any habitat gains, which remain high even at large expenditure levels.

## 4 Discussion

Over the coming decades, substantial financial investments will be made to restore freshwater biodiversity by reconnecting rivers, making it important to understand the returns of common heuristic frameworks for fish passage planning. We show that connectivity proxies, developed for anadromous fish species, can largely mitigate the potential problem of stranded investments as they lead to investment strategies where the most downstream barriers are prioritized. In contrast, adding benefit proxies (e.g., habitat quality proxies) to these heuristics may reverse those gains by eroding the relationship between a barrier’s prioritization ranking and its position in the watershed.

Benefit proxies are valuable for their flexibility, enabling managers to use readily-available spatial environmental data in fish passage restoration without needing to estimate complex functional relationships between environmental factors and fish populations. Since these relationships are often spatially dependent and difficult to obtain at a broad scale [42], proxies offer a practical alternative for incorporating habitat quality. Additionally, scoring formulations usually result from collaborative efforts involving multiple parties, e.g., state or local governments, tribes, and environmental groups [22], making proxies an accessible way to accommodate diverse preferences.

However, this flexibility comes at a cost when the goal is restoring high-quality anadromous fish habitat. Our results show that, when habitat quantity is the only restoration goal, connectivity proxies perform nearly as well as optimization approaches. Additionally, we demonstrate that including benefit proxies, constructed to promote investments in restoring access to high-quality spawning and rearing habitat, can surprisingly lead to *smaller* gains in high-quality habitat than when allocating investments in such a way that ignores habitat quality altogether.

Together, our results suggest there may be readily-achievable opportunities to improve on anadromous fish passage restoration programs using heuristic approaches, which are typically preferred due to their simplicity, accessibility, ease of explanation, ability to be rapidly deployed, and flexibility in incorporating real-world complexities [22,32,33]. Specifically, our results show that a modest redesign of commonly used score-and-rank heuristics can produce meaningful gains in prioritization performance. The key improvement is scaling habitat-quality attributes, normalized on a scale from 0 to 1, by habitat potential (the shift from SRpp to SRpa). When quality metrics for a barrier enter the score independently of the amount of habitat that the barrier represents, as in SRpp, barriers with favorable quality values are more likely to be ranked above downstream barriers, increasing the risk of stranded investments. By linking habitat quality to the extent of habitat that can be made accessible, SRpa reduces the likelihood of these stranded investments and steers investments towards removing barriers blocking large quantities of high-quality habitat.

SRpa also brings the heuristic closer to the management objectives implied by the underlying metrics. In most settings, the goal is to increase the amount of habitat embedded within natural land cover or other desirable conditions, not to maximize the sum of proxy metrics, such as the sum of the percent of natural surrounding habitat over all removal projects. Additionally, the approach of scaling normalized habitat-quality attributes by habitat potential is flexible and can easily be applied to a wide range of habitat quality attributes.

It is important to note that, in contrast to optimization approaches, SRpa does not entirely eliminate the possibility of stranded investments, which can happen when, e.g., barriers are clustered together and there are sharp increases in habitat quality when moving from downstream up. In this case, the clustering leads to similar values of habitat potential and upstream barriers with higher quality habitat can be assigned higher scores than downstream barriers on the same stream network. However, in our case study the SRpa approach performs remarkably well and, importantly, SRpa approach utilizes data inputs that managers already have. For example, with data on habitat potential and the percent of natural land cover upstream of a barrier, one can easily calculate the natural habitat potential. While the exact benefits of redesigning heuristics cannot be known without an optimization benchmark, more explicitly accounting for gains in high-quality habitat is a low-cost management change and is unlikely to decrease heuristic performance, making it a potentially useful improvement outside of our study system. In this way, managers can realize efficiency gains while avoiding the real and perceived costs of adopting exact optimization methods.

There are three important factors to consider when interpreting our work. First, our stylized SR approach is simply representative of scoring systems used in our study system and other systems. It is not identical to a scoring system used by any one particular entity. For example, our results are based on a scoring system incorporating four project attributes whereas entities in our study system incorporate anywhere from 6–13 metrics [21]. This simplification has allowed us to explore targeted research questions and generate tractable and generalizable results.

Second, we have not explored all sources of inefficiencies in anadromous fish passage restoration heuristics, even within our study system. In addition to using benefit proxies, it is common practice to classify habitat quality variables into discrete numerical values, such as a scale from 1 to 5. The functional relationship between the variable and the scale often does not correspond to the functional relationship between the variable and the objective, e.g., the variable and fish populations. [43] refer to this as a problem of “arbitrariness”. Future work should consider the impact of arbitrariness on returns to investments in improving fish passage.

Third, our analysis assumes that investment decisions are made by a central planner rather than several uncoordinated entities. The focus of our analysis is motivated by efforts towards a coordinated state-wide restoration strategy [44], however, [45] demonstrate the potential for large inefficiencies when uncoordinated actors apply optimization methods to fish passage restoration (heuristic planning is not considered). The result depends on the assumption that individual equally funded watershed managers act simultaneously and do not observe or predict restoration activities in neighboring watersheds. In reality, planning entities are often heterogeneous with respect to timelines and funding levels, have the ability to observe or predict restoration plans of neighboring entities, and face complications with holdout landowners that can prevent restoration activities at some sites altogether. Future work should consider the relative performance of heuristic approaches to fish passage restoration in the face of an array of coordination issues as well as solutions to coordination failures.

Finally, our analysis assumes that heuristic and optimization algorithms generate restoration plans that are then perfectly executed. In practice, score-and-rank approaches are often inputs to more complex decision processes that integrate other sources of information, such as local ecological knowledge. In this sense, outcomes from a score-and-rank exercise can guide further data collection and help determine high-level restoration strategies [22]. However, when heuristic rankings systematically prioritize stranded investments by failing to account for watershed connectivity, their practical utility is reduced and subsequent decision-making becomes more difficult.

As investments in fish passage restoration continue to grow worldwide, understanding how heuristic approaches translate into ecological outcomes is increasingly important. Our study shows that heuristic prioritization frameworks, when carefully designed, can guide a largely efficient allocation of restoration resources while remaining practical and accessible for on-the-ground decision-making. Identifying general principles that improve heuristic performance can simplify data-driven restoration decisions, reduce the risk of inefficient or stranded investments, and enhance the returns on investments in anadromous freshwater biodiversity.

## Supporting information

S1 File Supporting information for “Improving Restoration Heuristics to Support Anadromous Fish Passage,” containing Figures SI1–SI4 and Table SI1. (PDF)
