# Supplementary material for: Improving restoration heuristics to support anadromous fish passage
Source: PLoS One. 2026 Jun 3;21(6):e0348150. doi: 10.1371/journal.pone.0348150 (PMC13232858; doi:10.1371/journal.pone.0348150)
Supplement: S1 File — (PDF) [file pone.0348150.s001.pdf]

Supporting Information

1 Inefficient Spending

Stranded investments are one form of inefficient spending. Another form of inefficient spending is when resources are allocated to expensive projects projects, with the largest benefits, even though greater overall benefits could be achieved by funding several projects, each with smaller individual benefits (Figure SI1).

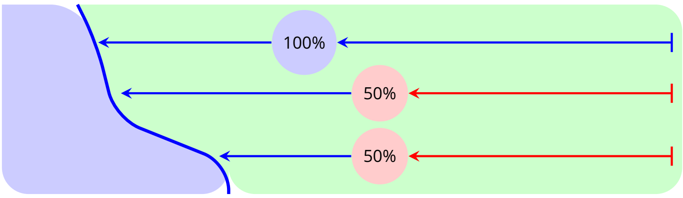

Figure SI1: Inefficient spending from a SR approach targeting total upstream habitat. Barriers are labeled their cost of restoration expressed as a percentage of the budget. The SR approach will restore the project with the largest amount of upstream habitat potential, which costs 100% of the budget. The optimal solution is to remove the two other barriers, which each cost 50% of the budget, for larger aggregate upstream habitat gains.

2 Variability in the performance of SRpa and SRpp

Results from our quality-focused analysis (Panel a; Figure 5) show that SRpa achieves roughly 93-95% of gains from optimization across all restoration goals. However, with alternative stream networks and cost distributions, the performance of SRpa may more greatly suffer from stranded investments and otherwise inefficient spending.

To assess the robustness of our result, we evaluate the performance of SRpa across 25 largely independent watersheds (HUC 8 regions) in our study system. As with our main results, we consider performance with a quantity weight of 0.3 and a budget level of 25% of the total cost of restoration. We find that the median relative performance, across watersheds, is only slightly lower than relative performance of restoration programs encompassing all watersheds, but that there can be outliers (Figure SI2). For example, the minimum relative performance of SRpa is 76% for total habitat, 70% for natural habitat, 76% for cool-water habitat, and 85% for species of interest. The relative performance of SRpp is less robust to the system of application with larger performance inter-quartile ranges and extreme minimum values (Figure SI2).

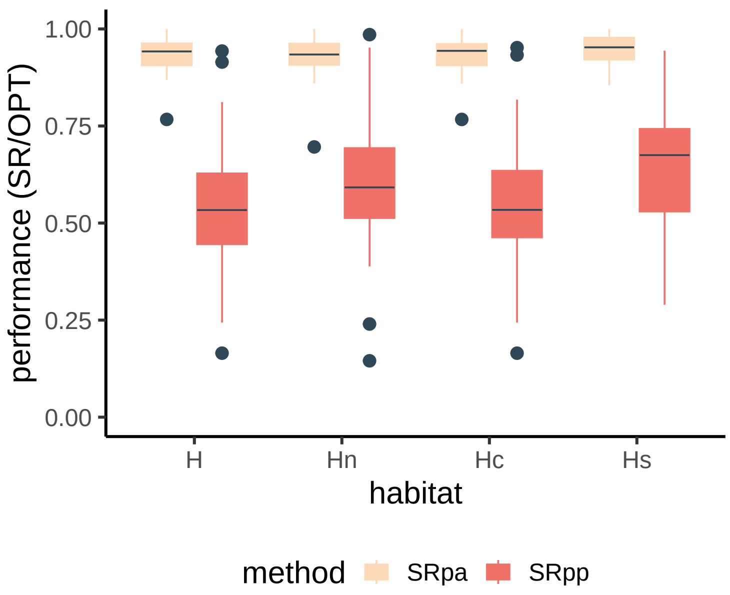

Figure SI2: Box plots show the distribution in the performance of SRpa and SRpp, relative to OPTaa. Boxes show the interquartile range of relative performance estimates, the line in the box shows the median, and points show outliers.

3 Robustness of SRpp Results

Our central results isolate a case when the weight placed on habitat quantity (α) is 0.3 and the budget, as a fraction of total cost (β), is equal to 0.25. We estimate 85% habitat gains can be realized through the optimal allocation investment funds equal to 25% of the cost of restoring all barriers. Figure SI3 presents the performance of SRpp under a range of α and β scenarios. Performance is measured as the percent gain from switching from SRpp to OPTaa and, for visualization purposes, is capped at 150%. For all outcomes, the gain from switching from SRpp to OPTaa is higher for smaller weights placed on habitat quantity (smaller α) and smaller budget levels (smaller β). When $\beta = \alpha$ = 0.05, switching from SRpp to OPTaa can lead to gains exceeding 276% for each of total habitat, natural habitat, and habitat for species of interest.

The results are also robust to penalties used to further adjust for connectivity. For example, many SR heuristics also contain penalty terms such that barrier scores are decreasing in the number of upstream barriers, downstream barriers, or both (Table SI1). Here we include a penalty term, λ on the normalized count of upstream and/or downstream barriers and compare outcomes to a SRpi approach without a penalty term. We explore high (λ = 0.5) and low (λ = 2.5) penalty values finding little to no improvements in the performance of SRp with low penalties and large reductions in performance with

high penalties (Figure SI4).

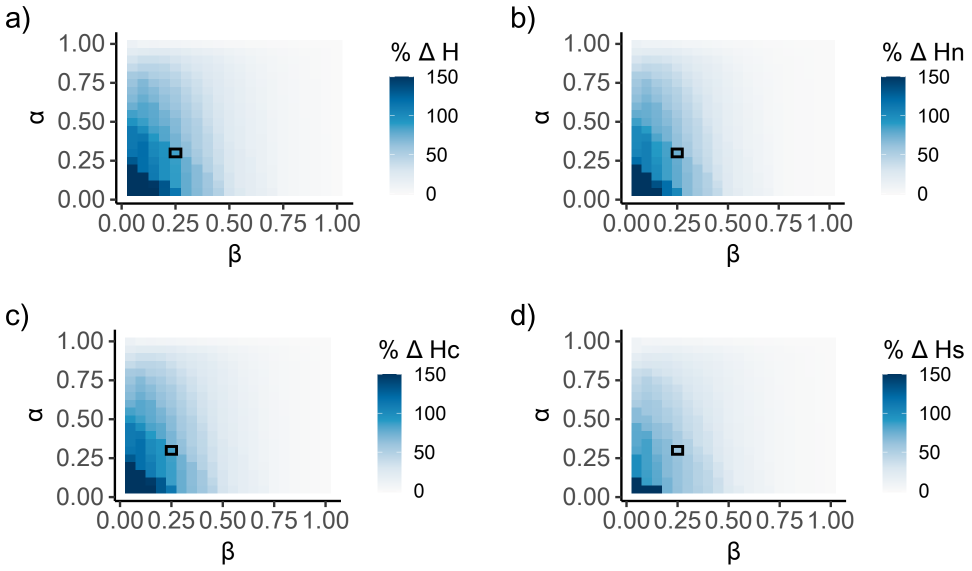

Figure SI3: The percent gain from switching from SRpp to OPTaa for outcomes: a) total habitat (H); b) natural habitat (Hn); c) cool-water habitat (Hc); d) and species of interest (Hs). Highlighted gridcells contain our central results.

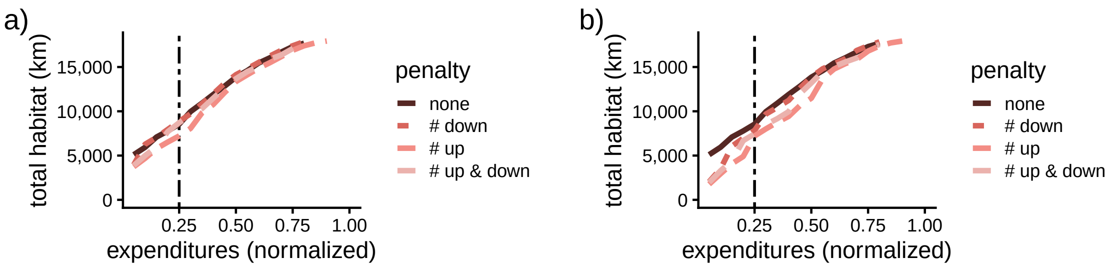

Figure SI4: Gains in total habitat quantity generated from SRpi heuristics that include low (Panel a) and high (Panel b) penalties for the (normalized) count of upstream barriers, the (normalized) count of downstream barriers, or both. Outcomes from the SRpi approach with no penalty (our central results) are included for comparison.

4 Scoring Systems in our Study Area and Elsewhere

Table SI1 documents multiple SR scoring systems that represent the framework we evaluate in this analysis. The SR systems documented here are taken from management reports and online tools and are not a comprehensive account of those that integrate both connectivity and habitat quality proxies. Table SI1 also records whether the SR scoring system uses a connectivity proxy, and a brief description of included proxy metrics capturing the three habitat quality attributes considered in our analysis: natural habitat, cool-water habitat, and habitat for target species of interest. Many of these scoring systems include penalties for the number of upstream barriers, downstream barriers, or both, and several other metrics. The use of penalties is recorded in Column 2 of Table SI1 and the robustness of our results to such penalties is explored (Figure SI4).

Table SI1: Examples of metrics included in documented SR heuristic scoring systems.

**Region (Ref.)**	**Connectivity**	**Natural**	**Cool Water**	**Target Species**
King County, Washington, USA (1)	Proxy, penalty	Forested & impervious (percent)	NA	Chinook or native Kokanee (binary)
Olympic Peninsula, Washington, USA (1)	Penalty only	Tree height, road density, riparian cover (units unk.)	Current & future stream temp. (dev. from 0° C)	Coho, steelhead, Chinook, sockeye, bull trout, resident trout (count)
Chehalis Basin, Washington, USA (1)	Proxy, penalty	Canopy cover (degrees) and tree height (meters)	Current & future stream temp. (dev. from 0° C)	Coho, steelhead, Chinook, sea-run cutthroat, and chum (count)
Thurston County, Washington, USA (1)	Proxy, penalty	Land use (units unk.)	NA	Any salmon species (binary)
City of Bellingham, Washington, USA (1)	Proxy only	Proximity to forest (units unk.)	NA	ESA listed species (binary)
Country of USA (2)	Proxy† only	Natural cover (percent)	NA	NA
Chesapeake Bay, USA (3)	Proxy, penalty	Natural cover (percent)	NA	NA
Northeastern states, USA (4)	Proxy, penalty	Natural cover (percent)	NA	Anadromous species (binary and count)
Brisbane, AU (5)	Penalty only	Non-intensive land use (percent)	NA	Listed, rare, and commercially valuable species (binary)

References denoted as (1) are documented in Burch et al. (2024); references denoted as (2) are documented in Southeast Aquatic Resources Partnership; references denoted as (3) are documented in Martin (2023); references denoted as (4) are documented in Martin and Levine (2017); references denoted as (5) are documented in Moore et al. (2018). Note that † represents a unique connectivity proxy not evaluated here.

References

Catalina A Burch, Sunny L Jardine, Connor Lewis-Smith, and Braeden Van Deynze. Who prioritizes what? a cross-jurisdictional comparative analysis of salmon fish passage strategies in Western Washington. *Conservation Science and Practice*, 6(5):e13102, 2024.

E.H. Martin. Chesapeake fish passage prioritization: An assessment of dams in the Chesapeake Bay watershed. Technical report, The Nature Conservancy, 2023.

EH Martin and J Levine. Northeast aquatic connectivity assessment project-version 2.0: Assessing the ecological impact of barriers on northeastern rivers. *The Nature Conservancy, Brunswick, Maine*, 2017.

Matt Moore, Jack McCann, and Trent Power. Greater Brisbane fish barrier prioritization. Technical report, Catchment Solutions, 2018.

Southeast Aquatic Resources Partnership. National aquatic barrier inventory and prioritization tool. URL `https://aquaticbarriers.org`.
